# Relapsing *Bacillus cereus* peritonitis in a patient treated with continuous ambulatory peritoneal dialysis

**DOI:** 10.1099/jmmcr.0.003400

**Published:** 2014-12-01

**Authors:** Anastasia Spiliopoulou, Evangelos Papachristou, Antigoni Foka, Fevronia Kolonitsiou, Evangelos D Anastassiou, Dimitrios S Goumenos, Iris Spiliopoulou

**Affiliations:** ^1^​ Department of Microbiology, School of Medicine, University of Patras, Patras, Greece; ^2^​ Department of Nephrology and Kidney Transplantation, School of Medicine, University of Patras, Patras, Greece

**Keywords:** *Bacillus cereus*, peritoneal dialysis, peritonitis, therapy

## Abstract

**Introduction:**

Peritonitis is a severe complication of peritoneal dialysis (PD) due to associated morbidity and mortality. Although *Bacillus cereus* is mostly considered as a contaminant, its role as a causative agent in a few cases of PD peritonitis has been documented. Peritonitis due to *B*. *cereus* has been associated with high rates of catheter removal and resistance to beta-lactam antibiotics.

**Case presentation:**

A case of relapsing peritonitis caused by *B. cereus* in a 69-year-old man with end-stage renal disease on continuous ambulatory PD for 3 years is described. *B. cereus* was recovered from the patient’s peritoneal fluid and was identified by phenotypic and molecular methods. The patient was treated, according to the susceptibility test, with tobramycin for 14 days. Cultures became sterile and the patient was discharged from hospital. Three days after discharge, the patient reported recurrence of abdominal pain and a new antibiotic regimen based on the previous culture results was initiated consisting of vancomycin and ciprofloxacin. The presence of *B. cereus* in the peritoneal fluid was confirmed, whereas repeated cultures for the next 15 days were positive. All *B. cereus* isolates produced biofilm. On day 16, the PD catheter was removed and the patient was transferred to haemodialysis. A review of previously reported cases is also presented.

**Conclusion:**

Since peritonitis is the most common cause of transition to haemodialysis, isolation of *B. cereus* from PD patients, even though rare, should not be considered as a contaminant. An appropriate antibiotic regimen and, whenever necessary, catheter removal should be applied.

## Introduction

Peritoneal dialysis (PD) is one of the main treatment modalities for patients with end-stage renal disease. Despite a series of technological innovations and improvements that have reduced overall infection rates, peritonitis prevalence ranges from 7.5 % to 40 % (Davenport, 2009). The spectrum of associated bacteria involves coagulase-negative staphylococci followed by *Staphylococcus aureus*, streptococci, enterococci, other Gram-positive organisms, *Pseudomonas aeruginosa* and other Gram negative bacteria, as well as fungi (Davenport, 2009; Nikitidou *et al*., 2012).

*Bacillus cereus* is a common cause of food poisoning; however, it has only occasionally been reported as the etiologic agent of other human infections, including peritonitis. A case of relapsing peritonitis caused by *B. cereus* in a patient on PD is described and a review of previously reported cases is presented.

## Case report

A 69-year-old man with end-stage renal disease on continuous ambulatory PD for 3 years was admitted with an 8-hour history of abdominal pain and fever of 38.5°C. The patient did not have any recent history of peritonitis or other infections. He did not complain of other gastrointestinal symptoms except abdominal pain and denied eating contaminated rice or food in bad condition. He lived in a rural area and did not adhere strictly to infection prevention recommendations. On admission, the peritoneal effluent was cloudy (white blood cell count 2375 μl^−1^, polymorphonuclear neutrophil cells 94 %), while Gram staining revealed the presence of large straight or slightly curved Gram-positive bacilli with square ends, singly or in short chains. Based on clinical signs and cell count, peritonitis was diagnosed and the patient was treated empirically with continuous intraperitoneal (IP) doses of cefuroxime [loading dose (LD): 1g, maintenance dose (MD): 250mg per 2 l exchange] and ceftazidime (LD: 1 g, MD: 250 mg per 2 l exchange), according to treatment guidelines suggested by the International Society for Peritoneal Dialysis (ISPD) (Li *et al*., 2010). Cultures in bottles were detected as positive (BacT/ALERT System, bioMérieux) and yielded Gram-positive bacilli with oval, centrally situated spores, which did not distort the bacillary form. Phenotypic identification as *B. cereus* was performed by BBL GP cards (bionumber 1315000165, Becton Dickinson Diagnostics), observation of irregular opaque colonies with rough matted surface surrounded by beta-haemolysis on blood agar plates, and a positive motility test. MICs of antimicrobials were determined by a gradient method (Etest, bioMérieux) according to the European Committee on Antimicrobial Susceptibility Testing (EUCAST) for *Staphylococcus* spp. and non-species related breakpoints (version 3.1, 2013, http://www.eucast.org; Lee *et al*., 2010). The isolate was susceptible to amikacin (2 mg l^−1^), gentamicin (1 mg l^−1^), tobramycin (1 mg l^−1^), ciprofloxacin (0.19 mg l^−1^), vancomycin (1 mg l^−1^), teicoplanin (0.094 mg l^−1^), linezolid (0.15 mg l^−1^), imipenem (0.094 mg l^−1^) and daptomycin (0.094 mg l^−1^), and resistant to ampicillin (>256 mg l^−1^), penicillin (>32 mg l^−1^), amoxicillin/clavulanic acid (>256 mg l^−1^), ceftazidime (>256 mg l^−1^), ceftriaxone (>32 mg l^−1^), aztreonam (>256 mg l^−1^) and sulfamethoxazole/trimethoprim (>32 mg l^−1^).

Identification to species level was confirmed by performing PCR using two pairs of universal primers for the 16S rRNA gene. The first pair consisted of the forward primer 16SrRNA1: 5′-TGCCAGCAGCCGCGGTAATAC-3′ and the reverse primer 16SrRNA2: 5′-CGCTCGTTGCGGGACTTAACC-3′, amplifying a 594 bp fragment at positions 10 129–10 722. The second pair of primers were forward 16SrRNA3: 5′-AGAGTTTGATCATGGCTCAG-3′ and reverse 16SrRNA4: 5′-GGYTACCTTGTTACGACTT-3′, amplifying a 413 bp fragment at positions 11 021–10 609 (Gatselis *et al*., 2006). Sequencing of the amplified products using the ABI PRISM 310 apparatus and comparison with existing universal microbial gene sequencing data (http://blast.ncbi.nlm.nih.gov/Blast.cgi) showed 100 % homology with the *B. cereus* 16S rRNA gene of strain FT9 (accession number CP008712.1), verifying the initial phenotypic identification.

In accordance to the susceptibility test, the antibiotic regimen was modified with replacement of ceftazidime by tobramycin (LD: 16 mg, MD: 8 mg per 2 l exchange) and treatment continued for a total of 14 days. Three days after the initiation of antibiotics, the patient’s clinical condition improved, peritoneal cell count decreased (35 μl^−1^), cultures became sterile and the patient was discharged from hospital after 15 days of antibiotic treatment.

Three days after discharge, the patient reported recurrence of abdominal pain; the effluent was cloudy and leukocytes increased to 505 μl^−1^ (polymorphonuclear cells 45 %). Accordingly, a new antibiotic regimen based on the previous culture results of peritoneal effluent was initiated consisting of IP vancomycin (LD: 1000 mg, MD: 50 mg per 2l exchange) and intravenous ciprofloxacin [200 mg twice daily (b.i.d.)]. New culture results confirmed the presence of *B. cereus* in the peritoneal fluid, which remained positive in repeated cultures for the next 15 days, despite continuous antibiotic treatment.

The PD catheter was surgically removed and the patient was transferred to haemodialysis through a right internal jugular venous catheter. Culture results of removed PD catheter confirmed the presence of *B. cereus*. All the recovered *B. cereus* isolates exhibited the same resistance phenotype to the antimicrobials tested. Furthermore, all were positive for biofilm formation by the use of 96-well microtitre plates and LB medium containing bactopeptone at 30 °C [background mean OD_595_ = 0.2±0.1; isolates’ mean OD_595_ = 1.4±0.2; *P*<0.001] (Auger *et al*., 2009).

## Discussion

For patients on PD, peritonitis is the most common cause of transition to haemodialysis, accounting for a significant morbidity and mortality ranging from 3.5 to 10 % (Odudu & Wilkie, 2011). In addition, peritonitis episodes have been implicated with loss of residual renal function, ultrafiltration failure and increased risk of encapsulating peritoneal sclerosis (Odudu & Wilkie, 2011). In many cases, despite the use of an appropriate antibiotic regimen, peritonitis relapses and catheter removal is often necessitated (Davenport, 2009). An important aspect in relapsing peritonitis is biofilm formation on PD catheters, as in our case (Nessim *et al*., 2012).

As a cause of PD-associated peritonitis, *B. cereus* has been recognized in seven previously reported cases accounting for eight patients, seven adults and one paediatric (Biasoli *et al*., 1984; Al–Wali *et al*., 1990; Al Hilali *et al*., 1997; Balakrishnan *et al*., 1997; Pinedo *et al*., 2002; Monteverde *et al*., 2006; Ruiz *et al*., 2006). The present case constitutes the ninth one, worldwide, and the first described in Greece. A review of cases published so far is shown in Table 1. In five cases patients underwent relapsing infections despite appropriate antibiotic treatment (Biasoli *et al*., 1984; Pinedo *et al*., 2002; Monteverde *et al*., 2006; Ruiz *et al*., 2006). In all but one case involving relapsing infections, the catheter was removed (Biasoli *et al*., 1984; Pinedo *et al*., 2002; Ruiz *et al*., 2006). According to ISPD guidelines, the focus should be on the preservation of the peritoneum rather than on saving the peritoneal catheter, whereas, the catheter should be removed in the case of relapsing peritonitis, refractory peritonitis, fungal peritonitis and refractory catheter infections (Li *et al*., 2010). The present case fulfils both criteria of relapsing as well as refractory peritonitis since in relapse, the effluent failed to clear after five days of appropriate antibiotic coverage (Li *et al*., 2010).

In general, most *B. cereus* isolates were resistant to beta-lactams (Turnbull *et al*., 2004; Luna *et al*., 2007; Uchino *et al*., 2012) and trimethoprim (Turnbull *et al*., 2004) and susceptible to ciprofloxacin, gentamicin and vancomycin (Turnbull *et al*., 2004; Luna *et al*., 2007; Uchino *et al*., 2012). The spectrum of effective antimicrobials includes fluoroquinolones, rifampicin, daptomycin, linezolid, and tigecycline (Luna *et al*., 2007). Susceptibility to erythromycin (Turnbull *et al*., 2004; Luna *et al*., 2007), clindamycin (Luna *et al*., 2007; Uchino *et al*., 2012) and tetracycline (Turnbull *et al*., 2004; Luna *et al*., 2007) varies, whereas, resistance to carbapenems has been described in bacteraemic cases and environmental isolates (Luna *et al*., 2007; Savini *et al*., 2009; Uchino *et al*., 2012). In the present case, the bacterium was resistant to the combination of cephalosporins initially administered as empiric therapy. As recommended, once culture and susceptibility results are available, antibiotic therapy must be adjusted (Li *et al*., 2010), and tobramycin was added in place of ceftazidime. By the patient’s improvement and effluent clearance, therapy continued for 2 weeks, as recommended for coagulase-negative staphylococcal and streptococcal peritonitis (Li *et al*., 2010). At the relapse, taking into account susceptibilities of the previously isolated pathogen and ensuring Gram-positive and Gram-negative coverage (Li *et al*., 2010), an alternative antibiotic combination consisting of IP vancomycin and intravenous ciprofloxacin was chosen. This combination has also been proposed in a recent study, as first line antibiotic therapy (Goffin *et al.*, 2004). The selected antibiotics failed to eradicate infection and catheter removal was necessitated. In the reported cases shown in Table 1, *B. cereus* peritonitis was cleared and the catheter was preserved in four cases. IP/intravenous (IV) vancomycin plus IP aminoglycoside (netilmicin or gentamicin) for three or 4 weeks was used in two cases (Balakrishnan *et al*., 1997; Al Hilali *et al*., 1997), teicoplanin for 3 weeks was used in a third case (Al–Wali *et al*., 1990), whereas, IP cefalotin and IP ceftazidime plus oral ciprofloxacin for 3 weeks was administered in the fourth case (Monteverde *et al*., 2006). One of the aforementioned combinations, IP vancomycin plus IP gentamicin, administered for 2 weeks, has led to relapse in another case (Pinedo *et al*., 2002). Although several antibiotic regimens have been tried with rather inadequate results, catheter removal led to resolution of the infection in all cases.

Although *B. cereus* peritonitis in patients on PD is very rare, when isolated, it should not be considered as a contaminant. Clinicians and clinical microbiologists must both give serious consideration to the significance of *B. cereus* isolation and design the best strategy, including an appropriate antibiotic regimen and whenever needed, catheter removal.

**Table 1.** Reported cases of PD-associated peritonitis caused by *B. cereus*: phenotypes, treatment and patient outcomes

**Author**	**Age, sex**	**Susceptibility**	**Resistance**	**Treatment**	**Outcome**
Biasoli *et al*. (1984)	71, M	Gentamicin	Cefotaxime	IP cefotaxime (100 mg l^−1^), on day 3 replacement by gentamicin (5 mg l^−1^); relapse catheter removal	Relapse, catheter removed
Al–Wali *et al*. (1990)	67, M	Teicoplanin	Aztreonam	Teicoplanin 200 mg for 3 weeks	Complete cure
Balakrishnan *et al*. (1997)	73, M	Vancomycin, netilmicin, teicoplanin, erythromycin	Penicillin	Starting doses of IV vancomycin 500 mg and netilmicin 150 mg, followed by continuous IP vancomycin (12.5mg l^−1^) and netilmicin (7.5 mg l^−1^) for 3 weeks	Complete cure
Al Hilali *et al*. (1997)	65, M	Vancomycin, teicoplanin, clindamycin, erythromycin	Piperacillin, cephalosporin	Vancomycin IV (1g) and IP gentamicin (LD:80 mg, MD 8 mg L^−1^) followed by vancomycin (1g) IV weekly for 4 weeks	Complete cure
Pinedo *et al*. (2002)	60, F	Vancomycin, erythromycin, cotrimoxazole	Cefuroxime, amoxicillin, penicillin	IP gentamicin (LD: 120 mg) and cefuroxime (LD: 1500 mg, MD: 500mg) for 2 weeks; relapse IP gentamicin (LD: 120 mg) and vancomycin (LD: 1 g, MD: 500 mg) for 2 weeks; relapse p.o. co-trimoxazole 480 mg for 2 weeks; relapse vancomycin for 6 weeks; relapse catheter removal	Relapse, catheter removed
Pinedo *et al*. (2002)	62, F	Vancomycin, erythromycin, tetracycline	Cefuroxime, penicillin, gentamicin	IP gentamicin (LD: 120 mg) and cefuroxime (LD: 1500 mg, MD: 500 mg) for 2 weeks; day 3 IP vancomycin (LD: 1 g, MD: 500 mg) for 2 weeks; relapse p.o. ciprofloxacin 500 mg b.i.d. for 6 weeks; relapse catheter removal	Relapse, catheter removed
Ruiz *et al*. (2006)	63, F	Vancomycin		IP gentamicin (LD: 80 mg, MD: 40 mg) and vancomycin (LD: 1 g, MD: 1 g every 5 days); day 3: gentamicin withdrawn and vancomycin continued for 2 weeks; relapse vancomycin 2 weeks; relapse vancomycin 2 weeks and catheter removal.	Relapse, catheter removed
Monteverde *et al*. (2006)	11, F	Gentamicin, ciprofloxacin, clindamycin, vancomycin, ceftazidime	Trimethoprim-sulfamethoxazole, penicillin	Intermittent IP vancomycin 30 mg kg^−1^ every 5 days and ceftazidime 15 mg kg^−1^ every 24 h for 21 days, oral nystatin as antimycotic prophylaxis; relapse IP cephalothin (15 mg kg^−1^) and ceftazidime (15 mg kg^−1^) plus oral ciprofloxacin 20mg kg^−1^, oral nystatin also given, total treatment lasted 21 days for cefalotin plus IP ceftazidime and oral ciprofloxacin	Relapse, no catheter removed
Present case, 2014	69, M	Vancomycin, teicoplanin, amikacin, gentamicin, tobramycin, ciprofloxacin, imipenem, cefoxitin Linezolid Daptomycin	Ampicillin, amoxicillin/clav, ceftazidime, ceftriaxone, aztreonam, trimethoprim-sulfamethoxazole	Continuous IP doses of cefuroxime (LD: 1 g, MD: 250 mg per 2 l exchange) and ceftazidime (LD: 1 g, MD: 250 mg per 2 l exchange); day 3 ceftazidime replaced by tobramycin (LD: 16 mg, MD: 8 mg per 2 l exchange); relapse vancomycin IP (LD: 1000 mg, MD: 50 mg per 2 l exchange) and ciprofloxacin IV (200 mg b.i.d.), catheter removal	Relapse, catheter removed

IV, intravenous; p.o., per os.

## Abbreviations

b.i.d.: twice daily
IP: intraperitoneal
ISPD: International Society for Peritoneal Dialysis
IV: intravenous
LD: loading dose
MD: maintenance dose
PD: peritoneal dialysis
